# Simulation of the nodal flow of mutant embryos with a small number of cilia: comparison of mechanosensing and vesicle transport hypotheses

**DOI:** 10.1098/rsos.180601

**Published:** 2018-08-08

**Authors:** Toshihiro Omori, Katja Winter, Kyosuke Shinohara, Hiroshi Hamada, Takuji Ishikawa

**Affiliations:** 1 School of Engineering, Tohoku University, Sendai Miyagi, Japan; 2 University of Potsdam, Brandenburg, Germany; 3 Tokyo University of Agriculture and Technology, Tokyo, Japan; 4 RIKEN Center for Developmental Biology, Kobe, Japan

**Keywords:** nodal flow, left–right asymmetry, boundary element method, fluid–structureinteraction

## Abstract

Left–right (L-R) asymmetry in the body plan is determined by nodal flow in vertebrate embryos. Shinohara *et al.* (Shinohara K *et al.* 2012 *Nat. Commun.* **3**, 622 (doi:10.1038/ncomms1624)) used *Dpcd* and *Rfx3* mutant mouse embryos and showed that only a few cilia were sufficient to achieve L-R asymmetry. However, the mechanism underlying the breaking of symmetry by such weak ciliary flow is unclear. Flow-mediated signals associated with the L-R asymmetric organogenesis have not been clarified, and two different hypotheses—vesicle transport and mechanosensing—are now debated in the research field of developmental biology. In this study, we developed a computational model of the node system reported by Shinohara *et al.* and examined the feasibilities of the two hypotheses with a small number of cilia. With the small number of rotating cilia, flow was induced locally and global strong flow was not observed in the node. Particles were then effectively transported only when they were close to the cilia, and particle transport was strongly dependent on the ciliary positions. Although the maximum wall shear rate was also influenced by ciliary position, the mean wall shear rate at the perinodal wall increased monotonically with the number of cilia. We also investigated the membrane tension of immotile cilia, which is relevant to the regulation of mechanotransduction. The results indicated that tension of about 0.1 μN m^−1^ was exerted at the base even when the fluid shear rate was applied at about 0.1 s^−1^. The area of high tension was also localized at the upstream side, and negative tension appeared at the downstream side. Such localization may be useful to sense the flow direction at the periphery, as time-averaged anticlockwise circulation was induced in the node by rotation of a few cilia. Our numerical results support the mechanosensing hypothesis, and we expect that our study will stimulate further experimental investigations of mechanotransduction in the near future.

## 1. Introduction

The vertebrate body plan has conserved left–right (L-R) asymmetry. For example, in the human body, the heart and spleen are arranged on the left side, whereas the gallbladder and most of the liver are located on the right [1]. The L-R asymmetric organogenesis is achieved in early vertebrate embryo development. At the stage of somitogenesis, several genes are asymmetrically expressed with respect to the L-R axis, which act as the trigger of L-R asymmetric organogenesis. The process of L-R asymmetric organogenesis can be divided into four steps [2]: (i) initial breaking of L-R symmetry might be involved in nodal flow in or near the node, (ii) transfer of L-R biased signals from the node to the lateral plate mesoderm, (iii) L-R asymmetric expression of signalling molecules, such as *Nodal* and *Lefty2*, in the lateral plate, and (iv) L-R asymmetric morphogenesis of the visceral organs is induced by these signalling molecules. Owing to recent biological experiments [1–3], it is found that fluid mechanics plays a crucial role in the first stage of L-R symmetry breaking.

In the early vertebrate embryo, e.g. 7.5 days for mice, there is an embryonic cavity at the ventral midline surface [3]. This cavity structure, the so-called node, is shaped as a roughly triangular depression with the apex pointed towards the anterior, and it is 50–100 μm in width and 10–20 μm in depth [1]. The node is covered by Reichert's membrane and filled with extraembryonic fluid [4]. The nodal pit surface is covered by a few hundred monociliated cells [1], and cilia can be observed at the nodal pit as rod-like protrusions that are 2–5 μm in length and 0.2–0.3 μm in diameter. The nodal cilia are tilted towards the posterior and produce leftward flow in the node by rotating in the clockwise direction, referred to as nodal flow. This cilia-driven nodal flow acts as the trigger expression of genes involved in L-R symmetry breaking, such as *Lefty2*, *Nodal* and *Pitx2* in the left lateral plate mesoderm [1,2,5]. Thus, nodal flow is crucial for the determination of L-R asymmetry, and there have been many investigations of the node system from a mechanical perspective.

To understand their mechanics, there have been a number of studies involving mechanical modelling of nodal cilia. Brokaw [6] developed a computational model of the nodal cilium using resistive force theory. In his model, active force, which is responsible for rotation, is regulated by sliding velocity and successfully simulates clockwise or anticlockwise rotation without the introduction of a symmetry-breaking mechanism. Hilfinger & Jülicher [7] also developed a nodal cilium model in which bending and twisting resistance of the cilium were determined as proportional to the curvature and the torsion, and internal displacement of the cytoskeletal microtubules was expressed in Fourier modes. Chen & Zhong [8] investigated nodal ciliary rotation with a three-dimensional finite element model. By changing the distribution function of the driving force, they concluded that, for smooth rotation, sliding velocity along the microtubule should be faster at the basal region and slower when it is close to the ciliary tip. Takamatsu *et al.* [9] performed an analytical investigation of the hydrodynamic interactions between two rotating cilia. The cilium was modelled as a rigid rod, and fluid viscous resistance was calculated by the boundary element method. The resulting phase lag between the two cilia was converged to *π*/2 rad, which agreed with experimental data. Although the ciliary rotation and synchronization were discussed intensively in these previous studies, many questions remain regarding flow-mediated signals for L-R symmetry breaking in the embryo.

For the flow-mediated signal, two expected scenarios were discussed in previous studies. One common hypothesis involves vesicle transport by leftward nodal flow, which is known as the vesicle transport hypothesis. Tanaka *et al.* [10] reported that fibroblast growth factor-induced lipid-enclosed parcels, so-called nodal vesicular parcels (NVPs), were released from nodal cells. NVPs contain morphogen proteins, such as sonic hedgehog and retinoic acid, and are expected to release them at the left periphery of the node. Smith *et al.* [11] numerically investigated particle transport using a slender body theory with a semi-infinite Stokeslet. They reported that, by rotating three cilia, particles were swept to the left and there was no continuous recovery rightward flow. If Reichert's membrane seals the nodal cavity, the counter-recovery rightward flow could be observed [4]. Smith *et al.* [12] also investigated geometric effects with regularized Stokeslets. In their paper, the node geometry was idealized to a triangular shape, but Reichert's membrane was taken into account. Particle trajectories within the enclosed domain showed leftward transport with unpredictable rotation near the cilia, whereas they drifted rightward distant from the cilia. Then, particles traced global circulation within the node and the left-specific transportation was not shown in the model. Counter-rightward flow in the enclosed domain was also reported by [13,14]. To the best of our knowledge, none of the theoretical and computational models can explain the vesicle transport hypothesis.

Mechanosensing with peripheral immotile cilia is an alternative asymmetry model, referred to as the mechanosensing hypothesis. McGrath *et al.* [15] reported that peripherally located cilia are immotile, but have the cation channel polycystin-2. Asymmetric calcium signalling appears at the left side of the node. They then proposed that L-R asymmetry is established by mechanosensing; centrally located motile cilia generate nodal flow, whereas cation channel-containing peripheral immotile cilia sense the nodal flow. Membrane tension is suggested to regulate the mechanosensing channel of the biological membrane [16–18]. Despite its biological importance, the membrane tension of immotile cilia has not been clarified, as there have been few fluid mechanical studies regarding mechanotransduction [19]. Thus, flow-induced membrane tension should be clarified to gain a better understanding of the mechanotransduction of immotile cilia.

Shinohara *et al.* [3] investigated the relationship between the number of rotating cilia and L-R marker gene expression using *Dpcd* and *Rfx3* mutant embryos at the four-to-six somite stage. Embryos with no or only one motile cilium did not show L-R asymmetric gene expression. However, embryos with between two and six rotating cilia exhibited normal L-R patterning, although only weak local flow was induced in the node. These observations suggest the presence of a highly sensitive system to sense very weak nodal flow. Sampaio *et al.* [20] also investigated nodal flow in zebrafish embryos with a small number of motile cilia. They reported that fluid flow with 30 cilia achieved 90% situs solitus, suggesting that strong fluid flow is not necessary to break L-R symmetry. In the case of wild-type embryos, it is difficult to evaluate the reliabilities of the two competing hypotheses [21]. In this study, we computationally reproduced the experiments of Shinohara *et al.* [3] to determine the quantitative threshold of nodal flow strength with a small number of cilia. Specifically, we computed the stress field in the node and associated membrane tension of immotile cilia and also calculated cilia-driven particle transportation within the node, which simulates the transport of NVPs. We then compared the mechanosensing and vesicle transport hypotheses and examined their feasibilities.

## 2. Governing equations and numerical method

In this study, we developed two computational models to investigate the embryonic node system: (i) modelling of nodal flow by prescribed ciliary motions with actual geometry of the node and (ii) the fluid–structure interaction model of peripheral immotile cilia to investigate flow-induced membrane tension. In the following subsections, we briefly explain the governing equations and numerical method for fluid mechanics and membrane mechanics.

### 2.1. Fluid mechanics

#### 2.1.1. Cilia-driven flow within the node.

**Figure 1.**
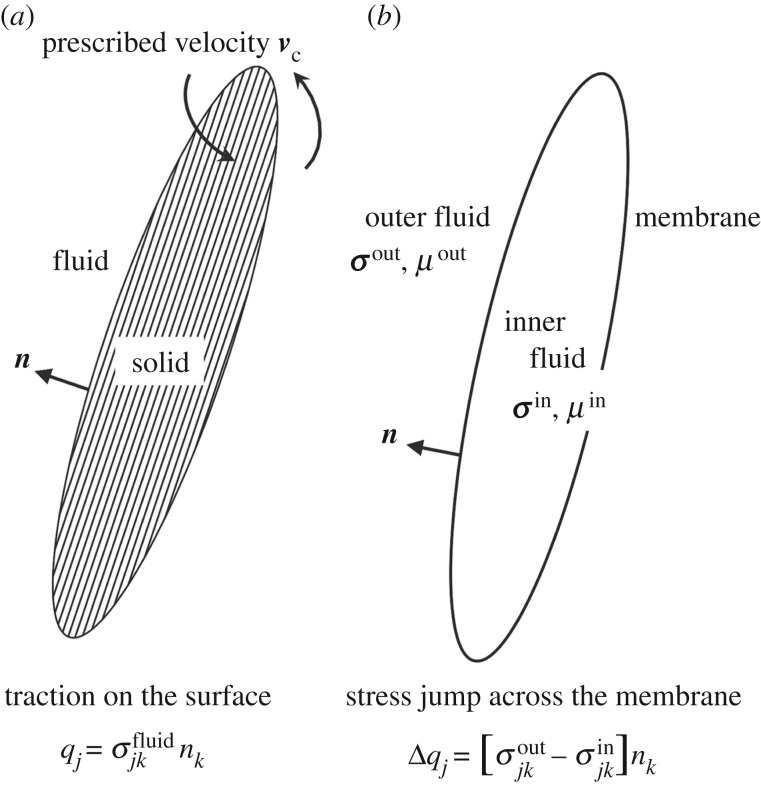
Schematics of the modelling of motile cilia and immotile cilia. (*a*) A motile cilium is modelled by a rigid rod and the surface traction ***q*** expresses the fluid traction. ***v***_c_ is the prescribed velocity, ***σ***^fluid^ is the fluid viscous stress tensor, and ***n*** is the outward unit vector. (*b*) An immotile cilium has a two-dimensional hyperelastic membrane. Stress jump across the membrane is expressed by Δ***q***. ***σ***^out^ and *μ*^out^ are the exterior fluid viscous stress and viscosity, and ***σ***^in^ and *μ*^in^ are those of interior fluid, respectively.

It is assumed that the embryonic node is covered by Reichert's membrane and that the enclosed cavity is filled with an incompressible Newtonian fluid. When we calculate the cilia-driven flow within the node, only motile cilia are placed in the node and immotile cilia are omitted. Owing to the small size of the cilia, the inertia effect of fluid motion can be neglected. Thus, we assume that the fluid flow is governed by the Stokes equation. We also assume that the motile cilia rotate as a rigid body and that velocity field ***v*** within the node can be determined by the following boundary integral equation:

2.1 $$v(x)=-\frac{1}{8\pi \mu }\int_{\mathrm{wall}}J(x,y)\cdot q(y)\;dS(y)-\frac{1}{8\pi \mu }\int_{\mathrm{cilia}}J(x,y)\cdot q(y)\;dS(y),$$

where ***x*** is an observation point, *μ* is the fluid viscosity, ***J*** is the Stokeslet and ***q*** = ***σ*** · ***n*** is the stress vector acting on the surface of the cilia or the nodal wall ***y*** (figure 1*a*). Here ***σ*** and ***n*** represent the fluid viscous stress and the outward unit normal vector, respectively. We note that the double-layer term of the boundary integral equation is deleted due to the boundary condition (no slip on the wall and rigid motion of motile cilia), and the equation is simplified to equation (2.1). Details about the boundary integral equation were presented previously [22].

To solve cilia-driven flow within the node, we model the motile cilium as a rigid rod of length *L* and radius *a*_c_. To mimic actual nodal cilia, the radius is set as *a*_c_/*L* = 0.05 throughout this study. Prescribed rotational velocity ***v***_c_(***y***, *t*) is applied to the model cilium, and the material point of the cilium ***y*** is updated by d***y***/d*t* = ***v***_c_(***y***, *t*). We set a constant angular velocity for all cilia and the cilia can rotate clockwise around the rotational axis. As the length of nodal cilia is determined by the somite stage of the embryo, we assume all cilia have the same length, *L*, and rotational frequency, *f*. We also assume a no-slip condition on the wall; ***v***(***y***;***y***∈*wall*) = 0, and equation (2.1) is solved with respect to unknown ***q*** using a boundary element method. The surfaces of the cilia and wall are discretized as triangular elements, and we have the following linear algebraic equation:

2.2 $$\left[\begin{array}{c} 0\\ v_{c} \end{array}\right]=\left[\begin{array}{cc} J_{\mathrm{ww}} & J_{\mathrm{cw}}\\ J_{\mathrm{wc}} & J_{\mathrm{cc}} \end{array}\right]\;\left[\begin{array}{c} q_{w}\\ q_{c} \end{array}\right].$$

The subscripts ‘w’ and ‘c’ indicate the surface of the wall and cilia, respectively. The matrix components *J*_ww_, *J*_cw_, *J*_wc_ and *J*_cc_ are computed from equation (2.1) using a Gaussian numerical integration scheme. The linear system of equation (2.2) is solved by lower upper (LU), and graphics processing unit (GPU) computing. These procedures continue over one period, as we assume periodic motions of the motile cilia. Once ***q*** is given, we can calculate the fluid velocity at any observation point, ***x***, from equation (2.1).

#### 2.1.2. Immotile cilia on an infinite plane wall.

In the mechanosensing hypothesis [15], immotile cilia located at the periphery of the node sense the flow by mechanical load, resulting in a left-sided signal. We also developed another computational model to simulate fluid–structure interaction of immotile cilia. To calculate flow-induced deformation of an immotile cilium, it is located on an infinite plane wall and simple shear flow is applied, instead of the nodal flow of equation (2.1).

We again assume that the fluid flow is governed by the Stokes equation, and the model of an immotile cilium is located on an infinite plane wall at *x*_3_ = 0. Flow due to the membrane deformation can be derived as follows:

2.3 $$\begin{array}{r} v(x)=v^{\infty }(x)-\frac{1}{8\pi \mu_{\mathrm{out}}}\int_{\mathrm{cilia}}J^{\prime }(x,y)\cdot \Delta q(y)\;dS(y)+\frac{1-\lambda }{8\pi }\int_{\mathrm{cilia}}v(y)\cdot T^{\prime }(x,y)\cdot n(y)\;dS(y), \end{array}$$

where ***v***^∞^ is the background flow, *λ*( = *μ*_in_/*μ*_out_) is the viscosity ratio of the inner and outer liquids, ***T***′ is the half-space Green's function of the double-layer potential, and Δ***q*** = [***σ***^out^ − ***σ***^in^] · ***n*** is the stress jump across the thin membrane (figure 1*b*). As we will discuss quasi-steady deformation of immotile cilia, the viscosity ratio *λ* is set to unity, and the double-layer term can be neglected. ***J***′ is the semi-infinite Stokeslet [23], which is given by

2.4 $$J^{\prime }(x,y)=J(x,y)-J(y,y^{\mathrm{IM}})+2y_{3}^{2}J^{D}(x,y^{\mathrm{IM}})-2y_{3}J^{\mathrm{SD}}(x,y^{\mathrm{IM}}),$$

where ***y***^IM^ = (*y*_1_, *y*_2_,  − *y*_3_) is a mirror image point of ***y***. ***J***^D^ is Green's function of a source doublet,

2.5 $$J_{ij}^{D}=(1-2\delta_{j3})\left(\frac{\delta_{ij}}{R^{3}}-\frac{3R_{i}R_{j}}{R^{5}}\right),$$

***J***^SD^ is Green's function of a Stokes doublet,

2.6 $$J_{ij}^{\mathrm{SD}}=(1-2\delta_{j3})\left(\frac{\delta_{ij}R_{3}-\delta_{i3}R_{j}+\delta_{j3}R_{i}}{R^{3}}-\frac{3R_{i}R_{j}R_{3}}{R^{5}}\right),$$

and ***R*** = ***x*** − ***y***^IM^. Once the velocity ***v*** is given, the membrane material point is updated by the second-order Runge–Kutta method.

### 2.2. Membrane mechanics

As ciliary membrane thickness is small compared with its length, the membrane of the immotile cilia is modelled as a two-dimensional hyperelastic material. As the membrane is supposed to be infinitely thin, the jump of viscous traction Δ***q*** is equal to the elastic load on the membrane. It is then related to the elastic tension tensor ***τ*** and the bending resistance ***q***_b_ in the interface by the membrane equilibrium equation

2.7 $$\nabla_{s}\cdot \tau +q_{b}+\Delta q=0,$$

where ∇_s_ is the surface gradient operator. The problem is closed with constitutive laws describing the elastic behaviour of the membrane.

Let ***Y*** and ***y***(***Y***, *t*) be a material point on the membrane in the reference and deformed states, respectively. The surface deformation gradient tensor ***F***_s_ is then given by

2.8 $$dy=F_{s}\cdot dY.$$

Local deformation of the membrane can be measured by the right Cauchy–Green tensor

2.9 $$C=F_{s}^{T}\cdot F_{s},$$

or by the Green–Lagrange strain tensor

2.10 $$e=\frac{1}{2}(C-I_{s}),$$

where ***I***_s_ is the tangential projection operator.

Two invariants of in-plane strain tensor ***e*** can be given by

2.11 $$I_{1}=\lambda_{1}^{2}+\lambda_{2}^{2}-2\;\mathrm{and}\;I_{2}=\lambda_{1}^{2}\lambda_{2}^{2}-1=J_{s}^{2}-1,$$

where *λ*_1_ and *λ*_2_ are the principal stretch ratios. The Jacobian *J*_s_ = *λ*_1_*λ*_2_ expresses the ratio of the deformed surface area to the reference surface area.

Assuming that the membrane is a two-dimensional isotropic hyperelastic material, the elastic stresses in an infinitely thin membrane are replaced by elastic tensions. The Cauchy tension ***τ*** can be related to an elastic strain energy per unit area *w*_s_(*I*_1_, *I*_2_)

2.12 $$\tau =\frac{1}{J_{s}}F_{s}\cdot \frac{\partial w_{s}(I_{1},I_{2})}{\partial e}\cdot F_{s}^{T}.$$

For the constitutive law, we employed the law of Skalak *et al.* [24]. The Skalak law can express strain-hardening behaviour and area incompressibility, and is often used for modelling of biological membranes. The surface strain energy function, *w*_s_ = *w*^SK^, and principal tensions in the membrane, *τ*_1_ and *τ*_2_ (*τ*_1_≥*τ*_2_), of the Skalak law are given by

2.13 $$w^{\mathrm{SK}}=\frac{G_{s}}{4}(I_{1}^{2}+2I_{1}-2I_{2}+CI_{2}^{2})$$

and

2.14 $$\tau_{1}=\frac{G_{s}\lambda_{1}}{\lambda_{2}}(\lambda_{1}^{2}-1+C\lambda_{2}^{2}(\lambda_{1}^{2}\lambda_{2}^{2}-1))\;(\mathrm{likewise}\;\mathrm{for}\;\tau_{2}),$$

where *G*_s_ is the surface shear elastic modulus and *C* is a dimensionless material coefficient that measures the resistance to area dilation. The area dilation modulus of the Skalak Law is given by *K*_s_ = *G*_s_(1 + 2*C*). By setting a large value of *C*, the area incompressibility of the membrane can be expressed. Usually, *C* = 10 is sufficiently high to express the area incompressibility [25]; therefore, we set *C* = 10 throughout this study.

The bending resistance of the membrane is also taken into account. The bending energy function of the lipid bilayer was derived by Zhong-can & Helfrich [26]. Using the first variation of the bending energy, the bending force density is given by

2.15 $$q_{b}=[E_{b}(2H+c_{0})(2H^{2}-2K-c_{0}H)+2E_{b}\Delta_{s}H]n,$$

where *E*_b_ is the bending modulus, *H* is the local mean curvature, *K* is the local Gaussian curvature, Δ_s_ is the Laplace–Beltrami operator and *c*_0_ is the spontaneous curvature of the membrane. The reference state is assumed to be a flat shape and *c*_0_ is set to zero in this study.

To couple fluid motions and membrane deformations, we use a finite element procedure for in-plane deformations of the membrane. By introducing an elastic membrane load balancing to the in-plane tension ***q***_p_ = ∇_s_ · ***τ***, a weak form of the equilibrium equation without the bending resistance is given by [27]

2.16 $$\int \hat{u}\cdot q_{p}\;dS=-\int \hat{\varepsilon }:\tau \;dS,$$

where $\hat{u}$ and $\hat{\varepsilon }$ are the virtual displacement and strain, respectively. The surface *S* in equation (2.16) indicates the median surface of the immotile cilia, and equation (2.16) is solved with respect to ***q***_p_ using a finite element method.

Stress jump across the membrane is determined by Δ***q*** + ***q***_p_ + ***q***_b_ = **0**, and it is coupled to equation (2.3). For details regarding the boundary element–finite element coupling method, refer to our previous study [25] or the electronic supplementary material of this paper.

### 2.3. Geometric model

**Figure 2.**
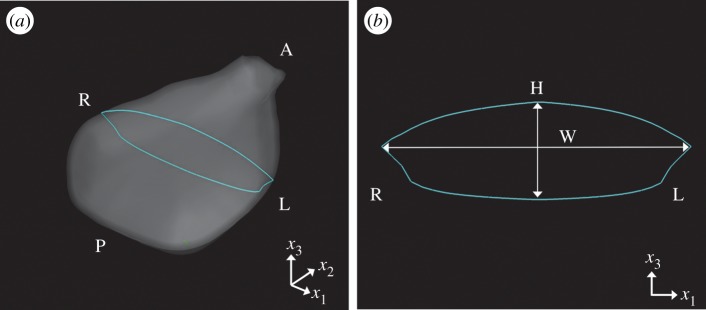
Computational model of the node. (*a*) Overview and (*b*) cross-section of the node, from the location indicated by the blue line in (*a*). Geometry of the node and positions of the cilia are taken from [3]. L and R indicate left and right directions of the embryo, while A and P are the anterior and posterior sides, respectively.

**Figure 3.**
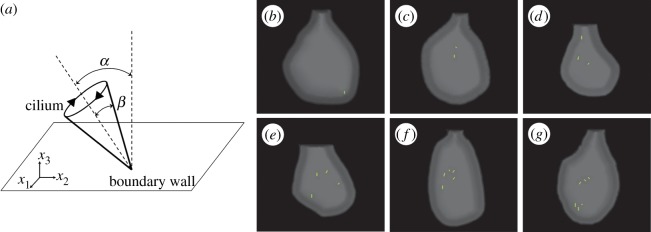
(*a*) Schematics of tilt angle *α* and open angle *β*. Tilt angle is defined as the angle between the rotational axis and the *x*_3_-axis, while *β* is equivalent to the cone angle. (*b*–*g*) Ciliary positions in the node with number of cilia = 1–6, which are taken from [3]. All cilia tilted towards the posterior direction (i.e. minus *x*_2_-direction).

Shinohara *et al.* [3] found that two rotating cilia are sufficient to break L-R symmetry, suggesting the existence of a highly sensitive system in the node to sense very weak nodal flow. To investigate such weak cilia-driven flow quantitatively, we developed a computational model of nodal flow based on the actual geometry of the embryonic node of Shinohara *et al.* [3]. Figure 2 shows one example of the computational model. In the figure, one cilium rotates in the node, which is shown in green. We traced two-dimensional nodal shapes of the experimental results of [3], then computationally reconstructed three-dimensional node geometries. The centreline, *s*, along with the anterior–posterior axis is defined as a bisector of the width, *W*, along the L-R axis. The height, *H*, is then determined by *H*(*s*)/*W*(*s*) = *k*. The constant, *k*, is determined as the maximum height corresponding to 20 μm, as the node depth is about 10–20 μm [1]. To mimic the cliff structure of the perinode, we used a hyperbolic tangent function for the side and base geometry. For Reichert's membrane, we used an ellipsoidal curve, as shown in figure 2*b*. The wall surface is discretized on a mesh of 3468 triangles, while the cilium model is discretized by 144 triangles. We developed one to six motile cilia models with different node geometries according to Shinohara *et al.* [3]. When we simulated the experiments of Shinohara *et al.*, the ciliary positions and phase difference among the cilia were determined from the experimental movies in [3]. When we compute with random ciliary positions, the initial phase difference is set randomly. For all simulations, the rotational angular velocity is fixed to 2*πf*. The rotational axis of the cilia is tilted towards the posterior side (figure 3*a*), and tilt and open angles of each cilium are also determined from the experimental movies with ellipsoidal fitting of rotational trajectories. To express the direction explicitly, hereafter, the Cartesian base vectors ***e***_1_, ***e***_2_ and ***e***_3_ correspond to the left, anterior and ventral directions, respectively. In table 1, typical numerical values are listed.

**Table 1.** Typical values of the variables used in this study.

viscosity *μ*	1 mPa s
length of cilia *L*	4 μm [1,11]
rotational frequency *f*	10 Hz [2]
shear elastic modulus *G*_s_	4 μN m^−1^ [28]
bending modulus *E*_b_	2 × 10^−19^ J [29]
tilt angle *α*	*π*/5 to 7*π*/30 rad
open angle *β*	*π*/30 to *π*/12 rad

## 3. Results and discussion

### 3.1. Velocity field in the node

**Figure 4.**
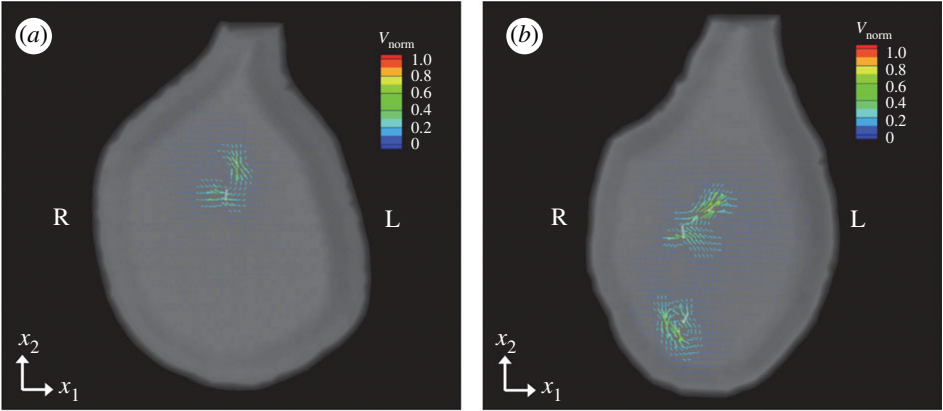
Velocity field of the node in the (*x*_1_, *x*_2_)-plane with a different number of cilia; (*a*) number of cilia = 2 and (*b*) number of cilia = 6. The height of the cross-section where the flow is visualized is set to *x*_3_ = *L*. The contour colour represents the velocity magnitude, which is normalized by the frequency *f* and the length of the cilia *L*. See the electronic supplementary material for movies [30].

We first investigated the flow field within the node. In this section and §§3.2 and 3.4, only motile cilia were placed in the node. Non-motile cilia at the periphery, discussed in §3.3, were omitted in these sections. In the Stokes regime, the fluid viscosity simply functions as a multiplier of traction. The fluid viscosity is then taken to be at unity, without loss of generality. Temporal fluid velocities with number of cilia = 2 and 6 are shown in figure 4; see also the electronic supplementary material, Movies. Fluid velocity is normalized by the cilium length *L*, and the frequency *f*, which are typically estimated as *L* = 4 μm and *f* = 10 Hz [1]. With fewer motile cilia, global flow was not observed in the cavity, but relatively large local vortical flow was seen around the rotating cilia. This local flow weakened sharply with distance and the recirculation area appeared only close to the motile cilia. Shinohara *et al.* [3] also mentioned that, when two motile cilia existed, local vortical flow appeared in the area close to the cilia, but the local flow decreased steeply with distance. In the experiment, they used a particle image velocimetry analysis to measure the velocity field in the node. By using confocal microscopy, the observation height was controlled to 5, 10, 15 and 20 μm from the apical cell surface in the node. They reported that the flow velocity close to the cilia was 1–1.7 μm s^−1^ with two or three rotating cilia. If we assume that the length of the cilia *L* = 4 μm and the rotational frequency *f* = 10 Hz, flow velocity at the *x*_3_ = 5 μm plane was about 2 μm s^−1^ at the maximum. Although it is hard to say that the observation height is exactly the same between the simulation and the experiment, our numerical result showed quantitative agreement with the experiment.

We also calculated cilia-driven circulation in the node. For simplicity, we focus on the *x*_2_-component of the circulation, as the rotational axis is parallel to the *x*_2_-axis. Global average circulation in the *x*_2_-axis is calculated as

**Figure 5.**
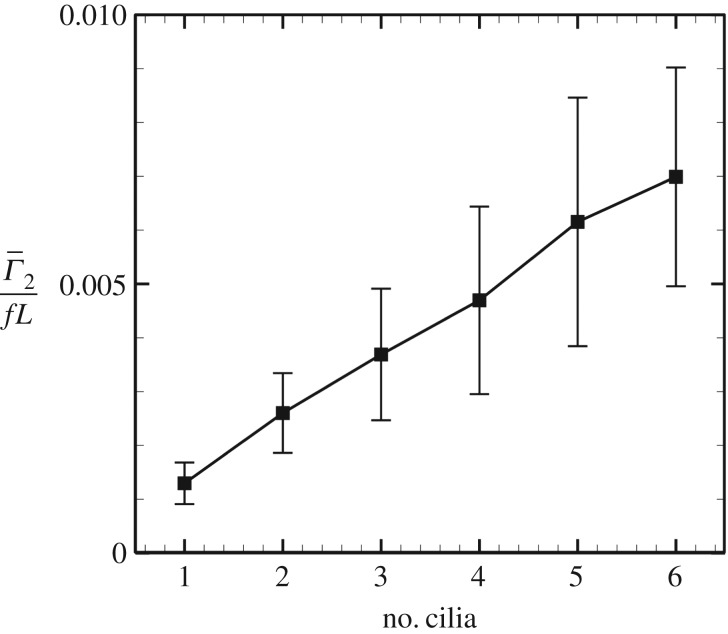
Time-averaged circulation as a function of number of cilia. *Γ*_2_ is the *x*_2_ component of the average circulation, *f* is the frequency and *L* is the length of the cilia.

3.1 $${\bar{\Gamma }}_{2}=\frac{1}{TL_{\mathrm{wall}}}\int_{0}^{T}\;\int_{\mathrm{wall}}\omega_{2}\;dS\;dt,$$

where *T* is the period of ciliary rotation (=1/*f*), $L_{\mathrm{wall}}\;(=\max x_{2}-\min x_{2})$ is the length of the node, *S* is the surface of the wall and *ω*_2_ is the *x*_2_-component of the vorticity. Then, the plus ${\bar{\Gamma }}_{2}$ indicates anticlockwise circulation viewing from the rear, while the minus indicates clockwise circulation. The results are shown in figure 5. To estimate the global average of circulation, we re-set the ciliary position and initial phase randomly, and each plot was averaged by *n* = 36 (6 different geometries × 6 different positions). The value increased monotonically with the number of rotating cilia, and it never had the negative sign, suggesting that weak anticlockwise circulation occurred in the node. Posterior-tilted rotating cilia cause leftward flow near the cilia, while counter-recovery flow should be generated distant from the cilia in the enclosed domain. Thus, time-averaged anticlockwise circulation occurred even when only local flow was induced by a few rotating cilia.

Shinohara *et al.* [3] also investigated the relationship between the positions of rotating cilia and L-R gene expression in *Dpcd* mutant embryos. Although local flow is strongly dependent on the ciliary position, normal L-R asymmetric expression of *Cerl2* and *Pitx2* was maintained regardless of position when two or more rotating cilia were present (*Cerl2* and *Pitx2* are expressed by the nodal flow, and can be seen as the genetic marker linked with L-R asymmetry). This suggested that flow-mediated signals for L-R asymmetry should be little influenced by the local flow. Flow-mediated signals may be sensed by perinodal cells [1]; as such, we investigated the mechanical fluid stress acting on perinodal cells.

### 3.2. Wall shear rate induced by nodal flow

The nodal flow generated by a small number of rotating cilia is localized and is strongly dependent on the ciliary position, although L-R asymmetry occurred regardless of the ciliary position. One possible candidate for the flow-mediated signal is mechanical stress on the perinodal cells. In this section, we discuss the cilia-induced stress field in the node.

To estimate the fluid viscous stress in the node, we first calculate the rate of strain,

3.2 $$E_{ij}(x)=\frac{1}{2}\left(\frac{\partial v_{i}}{\partial x_{j}}+\frac{\partial v_{j}}{\partial x_{i}}\right)=-\frac{1}{8\pi \mu }\int \left(-\frac{\delta_{ij}r_{k}}{r^{3}}+3\frac{r_{i}r_{j}r_{k}}{r^{5}}\right)q_{k}(y)\;dS(y),$$

where *r* = ∥***r***∥, ***r*** = ***y*** − ***x*** and surface *S* includes both the wall and the cilia. The strength of the mechanical stress acting on the perinodal wall is then estimated by wall shear rate, which is given by

3.3 $${\dot{\gamma }}_{w}(x;x\in \mathrm{perinodal}\;\mathrm{wall})\equiv 2\sqrt{E_{ij}E_{ji}}.$$

**Figure 6.**
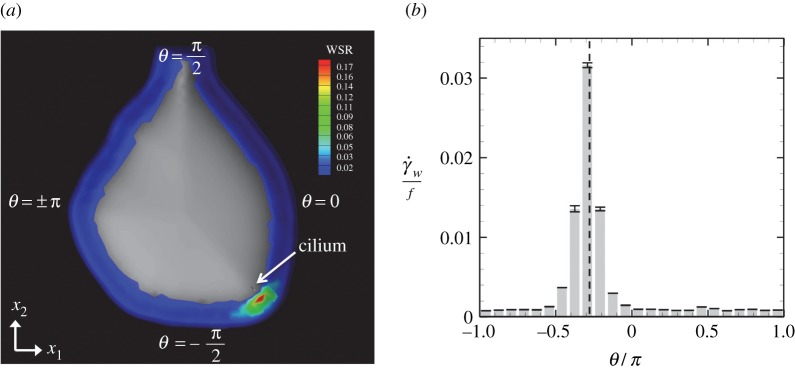
Wall shear rate acting on the perinodal cell generated by one cilium rotation. (*a*) Distribution of temporal wall shear rate. WSR indicates the wall shear rate ${\dot{\gamma }}_{w}/f$. (*b*) Spatial time-averaged wall shear rate ${\dot{\gamma }}_{w}$ with various *θ*. Error bars represent the time change of ${\dot{\gamma }}_{w}$. The vertical dotted line in the figure indicates the ciliary position.

The region of the perinodal wall is determined by the normal vector, $|n\cdot e_{3}|\leq \sqrt{3}/2$. The temporal wall shear rate acting on the perinodal wall is shown in figure 6*a*. Similar to the flow field, the region of high wall shear rate is local and the global wall shear rate is still weak when the number of cilia = 1. To investigate the distributions of the wall shear rate in detail, we introduced the orientation angle, *θ*, in the (*x*_1_, *x*_2_)-plane, as shown in figure 6*a*. The angle *θ* was discretized to 24 subdomains, and the wall shear rate was averaged in time and space in each subdomain; the resulting distribution is shown in figure 6*b*. The wall shear rate reached the maximum near the cilium, and the value decreased rapidly in *θ*-space.

**Figure 7.**
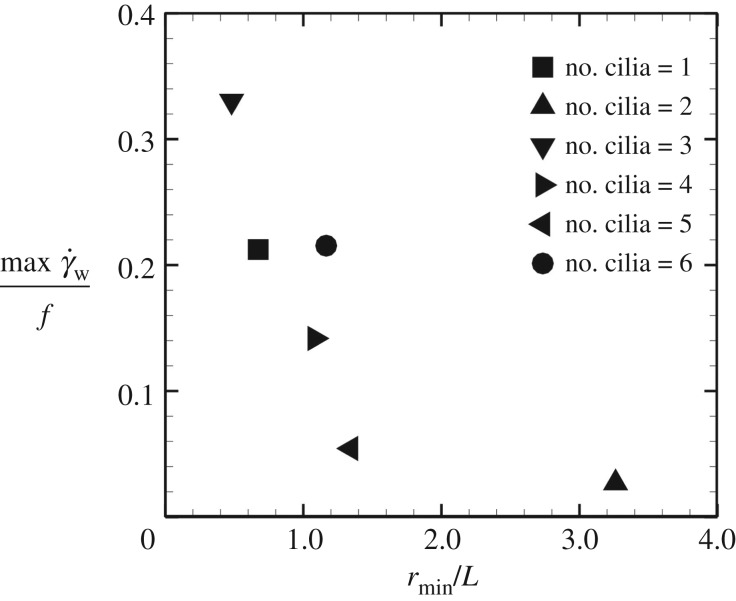
Maximum wall shear rate, $\max{\dot{\gamma }}_{w}$, as a function of the minimum distance between the cilia and the peripheral wall *r*_min_, normalized by frequency *f* and length of cilia *L*. The ciliary positions are the same as in figure 3.

We next investigated the spatio-temporal maximum wall shear rate with different numbers of motile cilia. The maximum wall shear rate was dependent on the distance between the cilia and the peripheral wall regardless of how many cilia are present. In figure 7, the maximum wall shear rate as a function of the minimum distance is shown. The distance $r_{\min}$ is defined as the instantaneous minimum distance between the peripheral wall, which was defined in equation (3.3), and the ciliary surface. As shown in figure 7, the value tended to decrease with distance, because the cilia-driven flow is local and the associated wall shear rate is also locally enhanced. These results suggest that the maximum value of wall shear stress is not adequate as the criterion of L-R symmetry breaking as it is influenced by the ciliary position.

**Figure 8.**
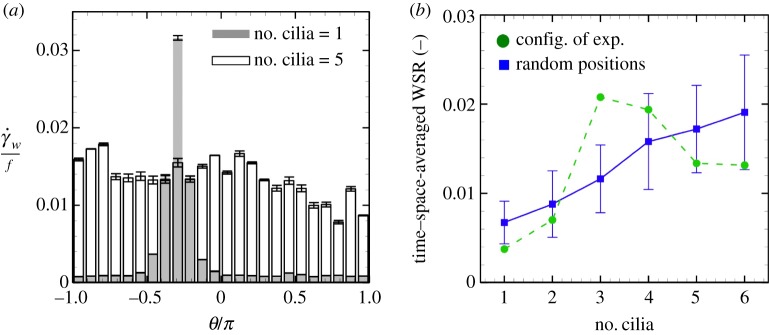
Mean wall shear rate. (*a*) Distributions of time-averaged wall shear rate ${\dot{\gamma }}_{w}$ with number of cilia = 1 and 5. (*b*) Time–space-averaged wall shear rate (WSR), which is normalized by the frequency *f*. Ciliary positions are obtained from [3] (green) or determined randomly (blue).

Although the maximum wall shear rate depends on the positions of the rotating cilia, the mean value showed a different tendency. Distributions of time-averaged wall shear rate with the number of cilia = 1 and 5 are shown in figure 8*a*. In this case, the maximum value for the number of cilia = 1 was larger than that for the number of cilia = 5. However, the average became larger with an increasing number of rotating cilia. We analysed the time–space-averaged wall shear rate with different numbers of cilia; the results are shown in figure 8*b*, indicated as green dots. The ciliary positions are determined from the experimental results of Shinohara *et al.* [3], which are equivalent to figure 3. The value tended to increase with the number of cilia, suggesting that the mean wall shear rate could be a candidate for the flow-mediated signal. We also see that the value became slightly larger when the numbers of cilia = 3 and 4. This is because the nodal domains were comparably small in these cases, as shown in figure 3. To increase generality, we re-set the ciliary position and initial phase randomly with various nodal domains, and estimated statistical averaged wall shear rate. The results are shown in figure 8*b*, depicted by blue squares, and each plot is averaged by *n* = 36 similar to figure 5. The average wall shear rate increased monotonically with the increasing number of cilia. This result suggests that the average wall shear stress is robust for the number of cilia and is little influenced by the ciliary position. Thus, mechanical stress fulfils the necessary conditions for the flow-mediated signal, and may be an adequate criterion. However, the estimated average wall shear rate is very small, with a value of about ${\dot{\gamma }}_{w}/f=O(10^{-2})$, and questions remain regarding how perinodal cells can sense such weak fluid shear stress. To clarify the mechanotransduction of perinodal immotile cells, we next investigated flow-induced membrane tension.

### 3.3. Membrane tension of immotile cilia

In the mechanosensing hypothesis [15], the perinodal immotile cilia sense the flow, resulting in a left-sided signal. The membrane tension plays an important role in mechanotransduction of biological cells. For example, the mechanosensitive calcium ion channels of *Escherichia coli* are activated by isotropic membrane tension [18]. In this section, we investigate the flow-induced membrane tension acting on the immotile cilia. We note that the immotile cilium is located on an infinite plane wall instead of the node with motile cilia. This is because the deformation of the immotile cilium is mainly induced by the local flow around the cilium, and the far-field fluid mechanics do not affect the results considerably.

To compute flow-induced membrane deformation, we introduce the capillary number, which represents the ratio of fluid viscous force and elasticity of the membrane, as determined by

3.4 $$\mathrm{Ca}=\frac{\mu \dot{\gamma }L}{G_{s}},$$

where *G*_s_ is the shear elastic modulus, *L* is the length of the cilia, *μ* is the fluid viscosity and $\dot{\gamma }$ is the shear rate. In the previous section, the average wall shear rate was given by ${\dot{\gamma }}_{w}/f=O(10^{-2})$. In particular, it reached a value of about ${\dot{\gamma }}_{w}/f=0.01$ with two cilia rotating in the node, which should be sufficient to break L-R symmetry if mechanical stress acted as a flow-mediated signal. Assuming a frequency of 10 Hz, the wall shear rate was estimated as ${\dot{\gamma }}_{w}=0.1\;s^{-1}$. As the membrane shear elastic modulus of the cilia is unclear, we assumed that it was the same as that of a red blood cell membrane, *G*_s_ = 4 × 10^−6^ N m^−1^ [28]. Using the fluid viscosity, *μ* = 1.0 × 10^−3^ Pa s and *L* = 4 × 10^−6^ m, the capillary number can be estimated as Ca = 10^−4^.

**Figure 9.**
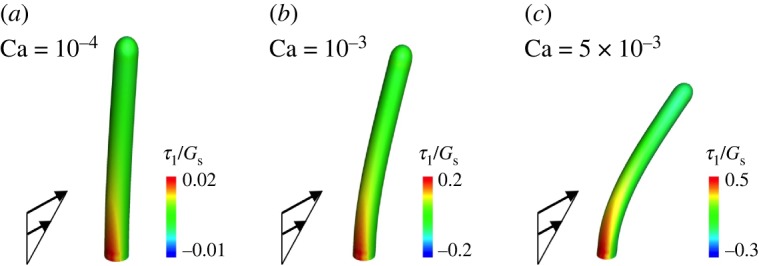
Membrane principal tension of non-motile cilia in shear flow. The shear stress is normalized by capillary number $\mathrm{Ca}=\mu \dot{\gamma }L/G_{s}$, where *μ* is the fluid viscosity, $\dot{\gamma }$ is the shear rate, *L* is the length of the cilium and *G*_s_ is the shear modulus. These are assumed as *μ* = 1 mPa s, *L* = 4 μm, and *G*_s_ = 4 μN m^−1^.

Deformation of the immotile cilia in shear flow with Ca = 10^−4^ is shown in figure 9*a*. Although the bending modulus of the ciliary membrane has not been clarified, we assumed that the bending modulus of the membrane equals that of a red blood cell membrane, which was estimated as *E*_*b*_ = 2 × 10^−19^ J [29]. Assuming that the length *L* = 4 × 10^−6^ m and the shear modulus *G*_s_ = 4 × 10^−6^ N m^−1^, the normalized bending modulus is given by *E*_b_/*L*^2^*G*_s_ = 3 × 10^−3^. The surface was discretized by 2160 triangle meshes. In order to calculate the membrane tension of immotile cilia, we used a semi-infinite Stokeslet. At the cellular scale, the flow around the boundary wall can be seen as linearized flow, and shear flow is useful to discuss the membrane tension. In addition, in our simulations, the typical time required for steady deformation was 10 times smaller than that for the rotational frequency. Therefore, the deformation could be assumed to be quasi-steady at any instant, though the deformation oscillates periodically. Because of this, we decided to use steady shear flow for the discussion. The cilia were slightly deformed in the flow direction, but did not show large deformation due to the weak flow. Although the bending deformation was relatively small, the membrane tension became larger at the base. As the boundary condition at the base was the fixed end, the load acting on the ciliary surface was integrated at the base to ensure the force and moment balanced. Accordingly, the tension could be enhanced at the base even with the small deformation limit, suggesting that membrane tension can be efficiently sensed by immotile cilia. The area of high tension was localized at the upstream side, whereas negative tension was observed at the downstream side of the bending deformation. The elongational tension would be important to open mechanosensitive channels in the ciliary membrane, and such localization may be helpful to sense the flow direction. As the problem setting of figure 9 is a single cilium placed on a flat wall under shear flow, a similar system can be found in some biological systems. For example, a primary cilium on an endothelial cell senses a fluid flow, and the mechanism may be explained by the mechanosensing as discussed in this study. Thus, the findings on membrane tension can be applied to other similar systems.

In the mechanosensing hypothesis, it is difficult to explain how immotile cilia sense the left or right. Anticlockwise circulation was generated in the node, and immotile cilia located at the left-peripheral wall tended to bend towards the plus *x*_3_-direction, while the right-peripheral cilia bent towards the minus *x*_3_-direction. As membrane tension was enhanced only at the upstream side, this difference may induce L-R asymmetry even when the magnitude of the wall shear stress is nearly isotropic in the node. If cytoskeletal proteins in the basal foot, such as microtubules and actin, composed anisotropic networks, similar to branchial cilia, bending rigidity of immotile cilia would be anisotropic and unidirectional circulation may also help to form L-R asymmetry.

**Figure 10.**
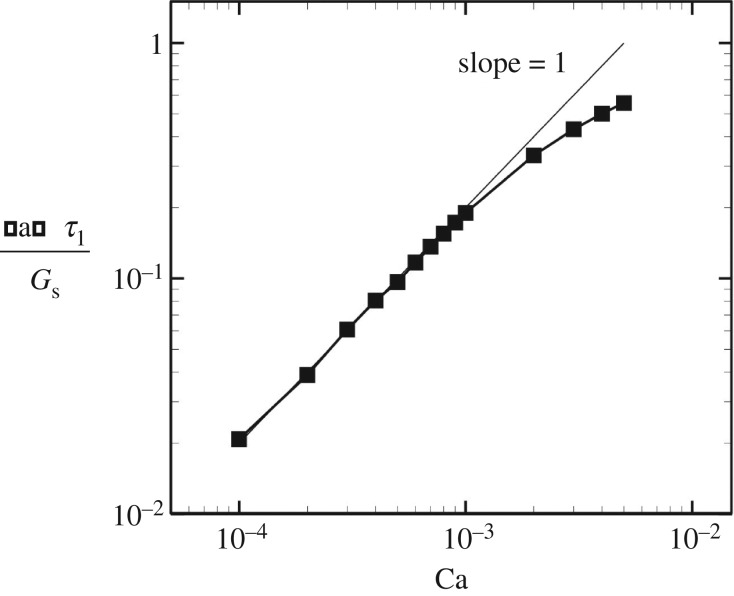
Maximum membrane tension as a function of the capillary number Ca. *τ*_1_ indicates the principal tension, and *G*_s_ is the shear elastic modulus of the membrane.

We also investigated large deformation of the immotile cilia by increasing the capillary number. The cilium showed large deformation and the membrane tension also increased with the capillary number. Similar to the small deformation limit, areas of high tension were localized at the upstream side, and negative tension occurred at the opposite side (figure 9*b*,*c*). The maximum tensions with various capillary numbers are shown in figure 10. The maximum tension was proportional to the capillary number for Ca≤10^−3^. In the previous experiment [3], two rotating cilia were sufficient to generate L-R asymmetry. When Ca = 10^−4^, which should be equivalent to the flow strength generated by two rotating cilia, the maximum tension became *τ*_1_/*G*_s_ = 0.02, as shown in the figure. Substituting *G*_s_ = 4 × 10^−6^ N m^−1^, it can be estimated as *τ*_1_ = 0.08 μN m^−1^. If mechanosensitive channels do play a role in flow-mediated L-R asymmetry, they may sense the tension at 0.1 μN m^−1^. This estimate would be influenced by the fluid viscosity, but not by *G*_s_. Further experimental studies of mechanosensitive channels are needed to gain a better understanding of mechanotransduction of immotile cilia.

Our numerical results support the mechanosensing hypothesis; however, the vesicle transport model should also be discussed. In the following section, we discuss cilia-driven particle transport.

### 3.4. Cilia-driven particle transport

The other important model is morphogen protein transport in the node. In this section, we investigate the probability density of left-transported particles with different numbers of cilia.

**Figure 11.**
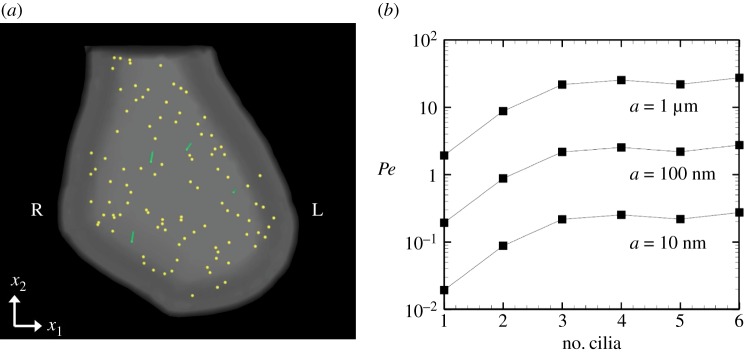
Cilia-driven particle transport in the node. (*a*) Initially, 100 particles are randomly distributed within the node. (*b*) Péclet number with different radii of the particle.

Before the particle trace calculation, we first estimated the Péclet number of particles of different radius, *a*. We assumed a temperature of 310 K and a fluid viscosity of 10^−3^ Pa s. When the radius of NVPs varied from 10 nm to 1 μm, the diffusion constant of the particles was estimated as 2.27 × 10^−14^≤*D* [m^2^ s^−1^]≤2.27 × 10^−12^ using the Stokes–Einstein equation. The Péclet number is defined as $Pe={\bar{\Gamma }}_{2}\ell /D$, where $\ell =\sqrt[{3}]{V}$ and *V* is the volume of the fluid domain. The estimated Péclet number is shown in figure 11*b*. In the case of *a* = 10 nm, the Péclet number is below 1 and the transport is diffusion dominant, whereas it is dominated by advection effects when *a* = 1 μm.

One hundred neutrally buoyant particles were initially distributed randomly within the node, as shown in figure 11*a*. Particle motion was then determined by the advection velocity calculated using equation (2.1) and diffusion due to Brownian motion. In the same manner as described previously [31], the positions of particles, ***x***^p^, were updated by

3.5 $$x^{p}(t+\Delta t)=x^{p}(t)+\int_{t}^{t+\Delta t}v^{p}(t^{\prime })\;dt^{\prime }+r^{B}(\Delta t),$$

where ***r***^B^ is the displacement due to Brownian motion, the variance of which is determined by the Péclet number. To discuss flow-dominant particle transportation, we used a *Pe* of *a* = 1 μm, throughout this study. When the distance, *d*, between the perinodal wall and the particle became smaller than *d*/*L* = 0.25, we assumed that the particle had reached the wall and tracking was stopped. If the particles reached the non-perinodal wall, they bounced back and the calculation was continued. The procedure was continued until all particles arrived at the perinodal wall or 9000 periods of the rotation.

**Figure 12.**
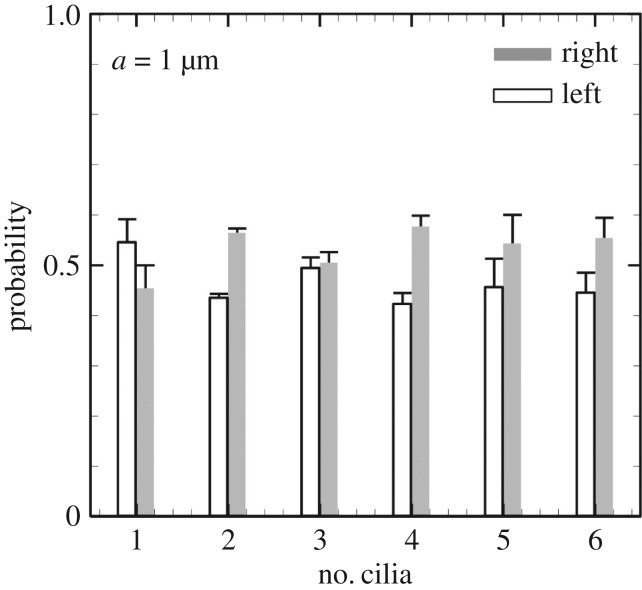
Ratio of left- and right-transported particles.

**Figure 13.**
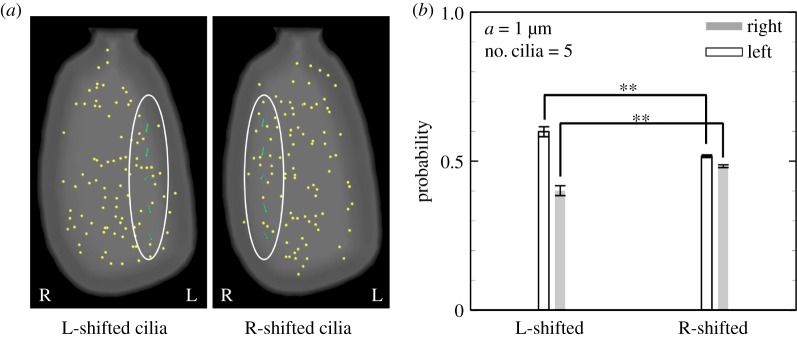
Effect of ciliary position on particle transport. (*a*) Five cilia are localized in the left or right side of the embryonic node, as indicated by the circle. (*b*) Probability of left- and right-transported particles with different ciliary positions.

The probability densities of peripherally transported particles are shown in figure 12. The results are summarized for three initial conditions. The probability was calculated as *P*_left_ = (particles reaching the left perinode)/(total number of particles reaching the perinode), and similarly for the right perinode. Left and right were determined by the sign of the *x*_1_-component of the final position. Particles were effectively advected when close to the motile cilia; however, the advection effect became significantly smaller when particles were far from the cilia (see the electronic supplementary material, Movies). Transportation was therefore dependent on the ciliary position, and the left transport probability was not enhanced by the number of cilia. To determine the effect of ciliary position, we compared the cilia aligned on the left and right (figure 13*a*). The probability was significantly altered by changing the ciliary position, as shown in figure 13*b*, despite the same number of rotating cilia in the node. According to the experimental results of Shinohara *et al.* [3], L-R asymmetric gene expression occurred regardless of ciliary position when there were more than two motile cilia, while the asymmetric gene expression was lost with one motile cilium regardless of the position of the cilium. Then, flow-mediated signals for the L-R asymmetry should be independent of the ciliary position. Ciliary position dependency was not adequate as a source of flow-mediated signals, and the vesicle transport hypothesis conflicted with the experimental results of Shinohara *et al.* [3]. In addition, anticlockwise circulation was generated in the enclosed domain even with a larger number of rotating cilia; thus, it may be difficult to achieve left-specific transportation of neutrally buoyant particles due to recovery rightward flow in the domain. When particles are heavier or lighter than the fluid, the transportation should be significantly influenced by the posture (prone/spine) of the embryo. Therefore, the vesicle transport hypothesis is again falsified.

## 4. Conclusion

In this study, we computationally reproduced the experiments of Shinohara *et al.* [3], and computed the cilia-driven particle transportation and stress field within the node, as well as associated membrane tension. Then, we compared the vesicle transport and mechanosensing hypotheses and discussed their feasibilities.

With a small number of cilia rotating in the node, the flow field was localized and strong global flow was not observed. Owing to this local flow, the particle transport was dependent on the ciliary position; therefore, the vesicle transport hypothesis conflicted with the experimental results of Shinohara *et al.* [3]. The maximum wall shear rate also showed strong ciliary position dependency, but the mean wall shear rate was little influenced by ciliary position and it was robust for the number of cilia. We also investigated the flow-induced membrane tension to determine the mechanotransduction of immotile cilia. To ensure the force and moment balance between the membrane tension and fluid stress, the membrane tension was increased at the base. The immotile cilia probably acted as a sensor of fluid stress even when the shear flow was weak. The high membrane tension area was localized at the upstream side of the base and the negative tension appeared at the opposite side. This localization of membrane tension is likely to be helpful in sensing the flow direction and producing L-R asymmetry, because time-averaged anticlockwise circulation was induced in the node.

Our numerical results support the mechanosensing hypothesis, but we still have questions about the mechanotransduction of immotile cilia, for example it is still unclear how immotile cilia sense the flow direction. We expect that our study will stimulate further experimental investigations of mechanotransduction in the near future.

## Supplementary Material

Supplemental document

## Data accessibility

Our data are deposited at Dryad: doi:10.5061/dryad.pd33j50 [30].

## References

[1] Hirokawa N, Okada Y, Tanaka Y 2009 Fluid dynamic mechanism responsible for breaking the left-right symmetry of the human body: the nodal flow. Annu. Rev. Fluid Mech. 41, 53–72. (10.1146/annurev.fluid.010908.165141)

[2] Hamada H 2002 Establishment of vertebrate left–right asymmetry. Nat. Rev. Genet. 3, 103–113. (10.1038/nrg732) 11836504

[3] Shinohara K *et al.* 2012 Two rotating cilia in the node cavity are sufficient to break left–right symmetry in the mouse embryo. Nat. Commun. 3, 622 (10.1038/ncomms1624) 22233632

[4] Nonaka S, Yoshiba S, Watanabe D, Ikeuchi S, Goto T, Marshall W, Hamada H 2005 De novo formation of left–right asymmetry by posterior tilt of nodal cilia. PLoS Biol. 3, e268 (10.1371/journal.pbio.0030268) 16035921 PMC1180513

[5] Capdevila J, Vogan K, Tabin C, Izpisua-Belmonte J 2000 Mechanisms of left–right determination in vertebrates. Cell 101, 9–21. (10.1016/S0092-8674(00)80619-4) 10778851

[6] Brokaw CJ 2005 Computer simulation of flagellar movement IX. Oscillation and symmetry breaking in a model for short flagella and nodal cilia. Cell Motil. Cytoskeleton 60, 35–47. (10.1002/cm.20046) 15573415

[7] Hilfinger A, Julicher F 2008 The chirality of ciliary beats. Phys. Biol. 5, 016003 (10.1088/1478-3975/5/1/016003) 18356578

[8] Chen D, Zhong Y 2015 A computational model of dynein activation patterns that can explain nodal cilia rotation. Biophys. J. 109, 35–48. (10.1016/j.bpj.2015.05.027) 26153700 PMC4572511

[9] Takamatsu A, Shinohara K, Ishikawa T, Hamada H 2013 Hydrodynamic phase locking in mouse node cilia. Phys. Rev. Lett. 110, 248107 (10.1103/PhysRevLett.110.248107) 25165968

[10] Tanaka Y, Okada Y, Hirokawa N 2005 FGF-induced vesicular release of sonic hedgehog and retinoic acid in leftward nodal flow is critical for left–right determination. Nature 435, 172–177. (10.1038/nature03494) 15889083

[11] Smith DJ, Gaffney EA, Blake JR 2007 Discrete cilia modelling with singularity distributions: application to the embryonic node and the airway surface liquid. Bull. Math. Biol. 69, 1477–1510. (10.1007/s11538-006-9172-y) 17473955

[12] Smith DJ, Smith AA, Blake JR 2011 Mathematical embryology: the fluid mechanics of nodal cilia. J. Eng. Math. 70, 255–279. (10.1007/s10665-010-9383-y)

[13] Cartwright JHE, Piro N, Piro O, Tuval I 2007 Embryonic nodal flow and the dynamics of nodal vesicular parcels. J. R. Soc. Interface 4, 49–55. (10.1098/rsif.2006.0155) 17015289 PMC2358960

[14] Smith AA, Johnson TD, Smith DJ, Blake JR 2012 Symmetry breaking cilia-driven flow in the zebrafish embryo. J. Fluid Mech. 705, 26–45. (10.1017/jfm.2012.117)

[15] McGrath J, Somlo S, Makova S, Tian X, Brueckner M 2003 Two populations of node monocilia initiate left-right asymmetry in the mouse. Cell 114, 61–73. (10.1016/S0092-8674(03)00511-7) 12859898

[16] Machemer H 1974 Frequency and directional responses of cilia to membrane potential changes in Paramecium. J. Comp. Physiol. 92, 293–316. (10.1007/BF00696617)

[17] Powers RJ, Roy S, Atilgan E, Brownell WE, Sun SX, Gillespie PG, Spector AA 2012 Stereocilia membrane deformation: implications for the gating spring and mechanotransduction channel. Biophys. J. 102, 201–210. (10.1016/j.bpj.2011.12.022) 22339856 PMC3260783

[18] Yoshimura K, Usukura J, Sokabe M 2008 Gating-associated conformational changes in the mechanosensitive channel MscL. Proc. Natl Acad. Sci. USA 105, 4033–4038. (10.1073/pnas.0709436105) 18310324 PMC2268802

[19] Chen D, Norris D, Ventikos Y 2011 Ciliary behaviour and mechano-transduction in the embryonic node: computational testing of hypotheses. Med. Eng. Phys. 33, 857–867. (10.1016/j.medengphy.2010.10.020) 21126903

[20] Sampaio P *et al.* 2014 Left-right organizer flow dynamics: how much cilia activity reliably yields laterality? Dev. Cell 29, 716–728. (10.1016/j.devcel.2014.04.030) 24930722

[21] Chen D, Norris D, Ventikos Y 2014 Chemosignalling, mechanotransduction and ciliary behaviour in the embryonic node: computational evaluation of competing theories. Proc. IMechE Part H: J. Eng. Med. 228, 465–476. (10.1177/0954411914531117) 24727590

[22] Pozrikidis C 1992 Boundary integral and singularity methods for linearized viscous flow. Cambridge, UK: Cambridge University Press.

[23] Blake JR 1971 A note on the image system for a Stokeslet in a no-slip boundary. Proc. Camb. Philos. Soc. 70, 303–310. (10.1017/S0305004100049902)

[24] Skalak R, Tozeren A, Zarda RP, Chien S 1973 Strain energy function of red blood cell membranes. Biophys. J. 13, 245–264. (10.1016/S0006-3495(73)85983-1) 4697236 PMC1484188

[25] Omori T, Ishikawa T, Barthés-Biesel D, Salsac A-V, Imai Y, Yamaguchi T 2012 Tension of red blood cell membrane in simple shear flow. Phys. Rev. E 86, 056321 (10.1103/PhysRevE.86.056321) 23214889

[26] Zhong-can OY, Helfrich W 1989 Bending energy of vesicle membranes: general expressions for the first, second, and third variation of the shape energy and applications to spheres and cylinders. Phys. Rev. A 39, 5280–5288. (10.1103/PhysRevA.39.5280) 9901091

[27] Walter J, Salsac A-V, Barthès-Biesel D, Tallec PL 2010 Coupling of finite element and boundary integral methods for a capsule in a Stokes flow. Int. J. Numer. Methods Eng. 83, 829–850. (10.1002/nme.2859)

[28] Hochmuth RM, Waugh RE 1987 Erythrocyte membrane elasticity and viscosity. Annu. Rev. Physiol. 49, 209–219. (10.1146/annurev.ph.49.030187.001233) 3551799

[29] Hwang WC, Waugh RE 1997 Energy of dissociation of lipid bilayer from the membrane skeleton of red blood cells. Biophys. J. 72, 2669–2678. (10.1016/S0006-3495(97)78910-0) 9168042 PMC1184464

[30] Omori T, Winter K, Shinohara K, Hamada H, Ishikawa T 2018 Data from: Simulation of the nodal flow of mutant embryos with small number of cilia: comparison of mechanosensing and vesicle transport hypotheses Dryad Digital Repository. (10.5061/dryad.pd33j50) PMC6124027 30225054

[31] Ishikawa T, Kajiki S, Imai Y, Omori T 2016 Nutrient uptake in a suspension of squirmers. J. Fluid Mech. 789, 481–499. (10.1017/jfm.2015.741)

