# Supplementary material for: Simulation of the nodal flow of mutant embryos with a small number of cilia: comparison of mechanosensing and vesicle transport hypotheses
Source: R Soc Open Sci. 2018 Aug 8;5(8):180601. doi: 10.1098/rsos.180601 (PMC6124027; doi:10.1098/rsos.180601)
Supplement: Supplemental document [file rsos180601supp1.pdf]

# Supplemental Document:
# Simulation of the nodal flow of mutant embryos with small number of cilia

Toshihiro Omori, Katja Winter, Kyosuke Shinohara, Hiroshi Hamada, and Takuji Ishikawa

## I. MOVIE CAPTIONS

**Movie 1**

Flow field with two rotating cilia. Flow magnitude was normalized by the frequency $f$ and the cilium length $L$.

**Movie 2**

Flow field with six rotating cilia. Flow magnitude was normalized by the frequency $f$ and the cilium length $L$.

**Movie 3**

Particle transport simulation. Neutrally buoyant 100 particles were randomly distributed at the initial.

**Movie 4**

Wall shear rate distribution during one period. The value was normalized by the frequency $f$.

## II. NUMERICAL METHOD

### A. Boundary element method

In this section, we explain a numerical method for fluid-structure interactions of immotile cilia. We assume that an immotile cilium is immersed in an incompressible Newtonian liquid with viscosity $\mu$ and density $\rho$. We also assume the cilium located on an infinite plane wall of $x_3 = 0$. Due to small size of the cilium, the Reynolds number is much smaller than unity (Re << 1), and the inertia effect can be negligible. The velocity field around the cilium can be derived as:

$$\begin{aligned}\boldsymbol{v}(\boldsymbol{x}) = \boldsymbol{v}^{\infty}(\boldsymbol{x}) - \frac{1}{8\pi\mu}\int_{cilia} \boldsymbol{J}'(\boldsymbol{x},\boldsymbol{y})\cdot\Delta\boldsymbol{q}(\boldsymbol{y})\,dS(\boldsymbol{y}) \\ +\frac{1-\lambda}{8\pi}\int_{cilia} \boldsymbol{v}(\boldsymbol{y})\cdot\boldsymbol{T}'(\boldsymbol{x},\boldsymbol{y})\cdot\boldsymbol{n}(\boldsymbol{y})\,dS(\boldsymbol{y}),\end{aligned} \tag{1}$$

where $\boldsymbol{v}^{\infty}$ is the background flow, $\lambda(=\mu_{in}/\mu)$ is the viscosity ratio of inner and outer liquids, $\boldsymbol{T}'$ is the half-space Green's function of double layer potential, and $\Delta\boldsymbol{q} = [\boldsymbol{\sigma}^{out} - \boldsymbol{\sigma}^{in}]\cdot\boldsymbol{n}$ is the stress jump across the thin membrane. Since we will discuss quasi-steady deformation of immotile cilia, the viscosity ratio $\lambda$ is set to unity, and the double layer term can be neglected. $\boldsymbol{J}'$ is the semi-infinite Stokeslet, which is given by

$$\boldsymbol{J}'(\boldsymbol{x},\boldsymbol{y}) = \boldsymbol{J}(\boldsymbol{x},\boldsymbol{y}) - \boldsymbol{J}(\boldsymbol{y},\boldsymbol{y}^{IM}) + 2y_3^2\boldsymbol{J}^{D}(\boldsymbol{x},\boldsymbol{y}^{IM}) - 2y_3\boldsymbol{J}^{SD}(\boldsymbol{x},\boldsymbol{y}^{IM}), \tag{2}$$

where $\boldsymbol{J}$ is the Stokeslet in an infinite domain,

$$J_{ij} = \frac{\delta_{ij}}{r} + \frac{r_i r_j}{r^3}, \tag{3}$$

$r = |\boldsymbol{r}|$, $\boldsymbol{r} = \boldsymbol{x} - \boldsymbol{y}$, $\boldsymbol{y}^{IM} = (y_1, y_2, -y_3)$ is a mirror image point of $\boldsymbol{y}$. $\boldsymbol{J}^{D}$ is the Green's function of a source doublet,

$$J_{ij}^{D} = (1-2\delta_{j3})\left(\frac{\delta_{ij}}{R^3} - \frac{3R_iR_j}{R^5}\right), \tag{4}$$

$\boldsymbol{J}^{SD}$ is the Green's function of a Stokes doublet,

$$J_{ij}^{SD} = (1-2\delta_{j3})\left(\frac{\delta_{ij}R_3 - \delta_{i3}R_j + \delta_{j3}R_i}{R^3} - \frac{3R_iR_jR_3}{R^5}\right), \tag{5}$$

and $\boldsymbol{R} = \boldsymbol{x} - \boldsymbol{y}^{IM}$.

The membrane surface $S$ is determined by two surface curvilinear coordinates $(\xi^1, \xi^2)$. The two base vectors are then defined by

$$\boldsymbol{a}_\alpha = \frac{\partial \boldsymbol{x}}{\partial \xi^\alpha}, \tag{6}$$

and the third base vector $\boldsymbol{a}_3 = \boldsymbol{n}$ is the unit outward normal vector. Greek indices indicate the two curvilinear coordinate (e.g. $\alpha = 1$, or 2). The associated contravariant base vector is also defined as: $\boldsymbol{a}^\alpha \cdot \boldsymbol{a}_\beta = \delta^\alpha_\beta$. The covariant and contravariant metric tensors are given by

$$a_{\alpha\beta} = \boldsymbol{a}_\alpha \cdot \boldsymbol{a}_\beta, \qquad a^{\alpha\beta} = \boldsymbol{a}^\alpha \cdot \boldsymbol{a}^\beta. \tag{7}$$

A differential surface element can be expressed by

$$dS = \sqrt{|a_{\alpha\beta}|} d\xi^1 d\xi^2, \tag{8}$$

where $|a_{\alpha\beta}|$ is the determinant of $a_{\alpha\beta}$.

To calculate the integral equation, we use a Gaussian numerical integration scheme with a linear interpolation function. When calculating any physical quantities inside a given element, the coordinates $(\xi^1, \xi^2)$ are replaced by the intrinsic coordinates in an isoparametric triangular element $(\eta^1, \eta^2)$ with the interval of [0,1]. The integral is then discretized by:

$$\int (\cdot) dS \simeq \sum_{el} \int_0^1 \int_0^{1-\eta^2} (\cdot) \sqrt{|a_{\alpha\beta}|} d\eta^1 d\eta^2. \tag{9}$$

When a observation point $\boldsymbol{x}$ is located near a source point $\boldsymbol{y}$, a special operation should be needed to avoid large numerical error arising from the singularity even if $\boldsymbol{x}$ does not coincide with $\boldsymbol{y}$. For the singular elements, which share the point $\boldsymbol{x}$, the polar coordinates $(\zeta, \psi)$ centered at the singular point $\boldsymbol{x}$ are introduced:

$$\int_0^1 \int_0^{1-\eta^2} (\cdot) \sqrt{|a_{\alpha\beta}|} d\eta^1 d\eta^2 = \int_0^{\frac{\pi}{2}} \int_0^{R(\psi)} (\cdot) \sqrt{|a_{\alpha\beta}|} \zeta d\zeta d\psi, \tag{10}$$

where $R(\psi) = 1/(\sin\psi + \cos\psi)$. Since the Jacobian $\zeta$ tends to zero as fast as the Euclidean distance $r$, the singularity is eliminated.

### B. Finite element method

As ciliary membrane thickness is small compared to its length, the membrane of immotile cilia is modelled as a two-dimensional hyperelastic material. Let $\boldsymbol{X}$ and $\boldsymbol{x}(\boldsymbol{X}, t)$ be a material point on the membrane in the reference and deformed states, respectively. Surface deformation gradient tensor $\boldsymbol{F_s}$ is then given by

$$\boldsymbol{F_s} = \boldsymbol{a}_\alpha \otimes \boldsymbol{A}^\alpha, \tag{11}$$

where $\boldsymbol{A}^\alpha$ are the curvilinear base vector in the undeformed state. Local deformation of the membrane can be measured by the right Cauchy-Green tensor

$$\begin{aligned} \boldsymbol{C} &= \boldsymbol{F_s}^T \cdot \boldsymbol{F_s} \\ &= a_{\alpha\beta} \boldsymbol{A}^\alpha \otimes \boldsymbol{A}^\beta, \end{aligned} \tag{12}$$

or by the Green-Lagrange strain tensor

$$\begin{aligned} \boldsymbol{e} &= \frac{1}{2} (\boldsymbol{C} - \boldsymbol{I_s}) \\ &= \frac{1}{2} (a_{\alpha\beta} - A_{\alpha\beta}) \boldsymbol{A}^\alpha \otimes \boldsymbol{A}^\beta, \end{aligned} \tag{13}$$

where $\boldsymbol{I_s}$ is the tangential projection operator.

Two invariants of in-plane strain tensor $\boldsymbol{e}$ can be given by

$$I_1 = \lambda_1^2 + \lambda_2^2 - 2 = a_{\alpha\beta} A^{\alpha\beta} - 2, \;\; I_2 = \lambda_1^2 \lambda_2^2 - 1 = J_s^2 - 1 = |a_{\alpha\beta}||A^{\alpha\beta}| - 1, \tag{14}$$

where $\lambda_1$ and $\lambda_2$ are the principal stretch ratios. The Jacobian $J_s = \lambda_1\lambda_2$, expresses the ratio of the deformed to the reference surface areas.

Assuming that the membrane is a two-dimensional isotropic hyperelastic material, the elastic stresses in an infinitely thin membrane are replaced by elastic tensions. The Cauchy tension $\boldsymbol{\tau}$ can be related to an elastic strain energy per unit area $w_s(I_1, I_2)$:

$$\boldsymbol{\tau} = \frac{1}{J_s}\,\boldsymbol{F_s}\cdot\frac{\partial w_s(I_1,I_2)}{\partial \boldsymbol{e}}\cdot\boldsymbol{F_s}^T, \tag{15}$$

or its contravariant representation is given by

$$\tau^{\alpha\beta} \;=\; \frac{2}{J_s}\frac{\partial w_s}{\partial I_1}A^{\alpha\beta} + 2J_s\frac{\partial w_s}{\partial I_2}a^{\alpha\beta}. \tag{16}$$

In order to develop the finite element formulation, the membrane mechanical problem can be stated as follow:

$$\int \hat{\boldsymbol{u}}\cdot\boldsymbol{\Delta q}\;dS = \int \hat{\boldsymbol{\varepsilon}} : \boldsymbol{\tau}\;dS, \tag{17}$$

where $\boldsymbol{\Delta q}$ is the stress jump across the membrane, and $\hat{\boldsymbol{u}}$, $\hat{\boldsymbol{\varepsilon}}$ are the virtual displacement and strain, respectively. For the discretization, we define the shape function $N$ on each three nodal point:

$$\begin{aligned} N^{(1)} &= 1-\eta^1-\eta^2, \\ N^{(2)} &= \eta^1, \\ N^{(3)} &= \eta^2. \end{aligned} \tag{18}$$

Using the Galerkin method, the left-hand side of (17) becomes

$$\int \hat{\boldsymbol{u}}\cdot\boldsymbol{\Delta q}\;dS = \sum_{el}\hat{u}_I^{(p)}\left(\int_0^1\int_0^{1-\eta^2} N^{(p)}N^{(q)}\sqrt{|a_{\alpha\beta}|}d\eta^1 d\eta^2\right)\Delta q_I^{(q)}, \tag{19}$$

where subscript $I$ indicates the $I$-th Cartesian component, and $(p)$ suggests the quantity at the node $p$. Using a similar procedure, the right hand side of (17) becomes

$$\int \hat{\boldsymbol{\varepsilon}} : \boldsymbol{\tau}\;dS = \sum_{el}\int_0^1\int_0^{1-\eta^2}\hat{\varepsilon}_{\alpha\beta}\tau^{\alpha\beta}\sqrt{|a_{\alpha\beta}|}d\eta^1 d\eta^2. \tag{20}$$

The tensor $\hat{\varepsilon}_{\alpha\beta}$ is related to the covariant representation of $\hat{\boldsymbol{u}}$:

$$\hat{\varepsilon}_{\alpha\beta} = \frac{1}{2}\left(\frac{\partial \hat{u}_\alpha}{\partial \eta^\beta} + \frac{\partial \hat{u}_\beta}{\partial \eta^\alpha} - 2\Gamma^i_{\alpha\beta}\hat{u}_i\right), \tag{21}$$

where $\Gamma$ is the Christoffel symbol. The covariant component of the virtual displacement must now be expressed in terms its Cartesian components:

$$\hat{u}_i = a_i^I\hat{u}_I = N^{(p)}a_i^I\hat{u}_I^{(p)}, \tag{22}$$

where $a_i^I$ is the Cartesian component of $\boldsymbol{a}_i$. The virtual strain tensor can be written as

$$\hat{\varepsilon}_{\alpha\beta} = \hat{u}_I^{(p)}\chi_{\alpha\beta}^{(p)I}, \tag{23}$$

where

$$\chi_{\alpha\beta}^{(p)I} = \frac{1}{2}\frac{\partial N^{(p)}}{\partial \eta^\beta}a_\alpha^I + \frac{1}{2}\frac{\partial N^{(p)}}{\partial \eta^\alpha}a_\beta^I + N^{(p)}\frac{\partial a_\alpha^I}{\partial \eta^\beta} - \Gamma^i_{\alpha\beta}N^{(p)}a_i^I. \tag{24}$$

The right hand side of (17) is then given by

$$\int \hat{\boldsymbol{\varepsilon}} : \boldsymbol{\tau}\;dS \;=\; \sum_{el}\hat{u}_I^{(p)}\int_0^1\int_0^{1-\zeta^1}\chi_{\alpha\beta}^{(p)I}\tau^{\alpha\beta}\sqrt{|a_{\alpha\beta}|}d\eta^1 d\eta^2. \tag{25}$$

Using (19) and (25), we finally have the discrete equilibrium equation, which is solved by a biconjugate gradient method.

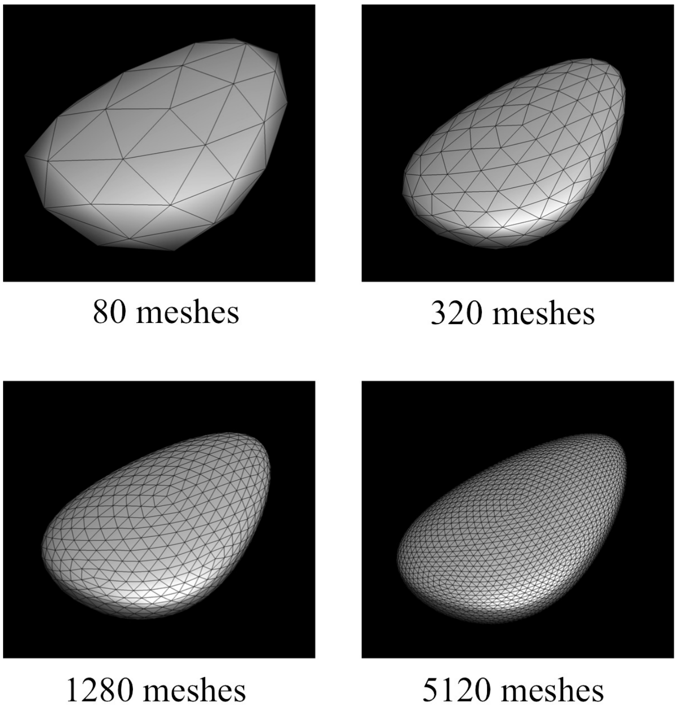


FIG. 1: Ideal node geometry discretized with various number of meshes.

### C. Mesh convergence

We checked the mesh convergence with ideal geometry of the node (cf. Fig.1). The node surface was discretized by 80, 320, 1280, and 5120 triangle meshes, and we calculated time-space average wall shear rate (WSR). The convergence is evaluated by

$$diff = \frac{|WSR - WSR_{5120}|}{WSR_{5120}},$$

where $WSR_{5120}$ is the reference wall shear rate calculated by the fine mesh (5120 triangles). The result is shown in Fig.2. We see that the difference becomes less than 3% when we use 1280 meshes. In the main text, the node is discretized by 3000 or more fine meshes, which should be large enough for the mesh convergence.

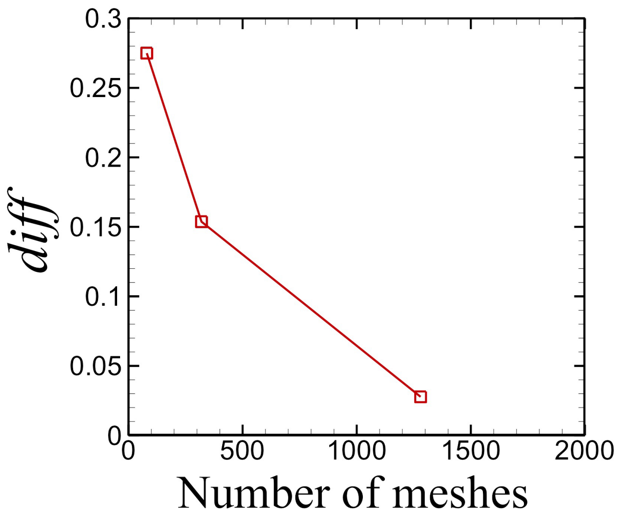


FIG. 2: Mesh convergence by calculating wall shear rate.
